# Application of Clinical Blood Metabogram to Type 2 Diabetes Mellitus

**DOI:** 10.3390/metabo14030168

**Published:** 2024-03-18

**Authors:** Petr G. Lokhov, Elena E. Balashova, Oxana P. Trifonova, Dmitry L. Maslov, Ekaterina A. Shestakova, Marina V. Shestakova, Ivan I. Dedov

**Affiliations:** 1 Institute of Biomedical Chemistry, 10 Building 8, Pogodinskaya Street, 119121 Moscow, Russia; balashlen@mail.ru (E.E.B.); oxana.trifonova@gmail.com (O.P.T.); dlmaslov@mail.ru (D.L.M.); 2 Endocrinology Research Centre, 11 Dm. Ulianova Street, 117292 Moscow, Russia; katiashestakova@mail.ru (E.A.S.); dedov@endocrincentr.ru (I.I.D.)

**Keywords:** metabogram, metabolomics, blood, diagnostics, mass spectrometry, clinical blood tests, personalized metabolomics, diabetes mellitus, impaired glucose tolerance

## Abstract

The clinical blood metabogram (CBM) was developed to match a tailored analysis of the blood metabolome to the time, cost, and reproducibility constraints of clinical laboratory testing. By analyzing the main blood metabolite groups, CBM offers clinically relevant information about the intake of low-molecular substances into the organism, humoral regulation, liver function, amino acid level, and the lipid and carbohydrate metabolism. The purpose of this work was to investigate the relevance of using the CBM in patients with diabetes mellitus. For this, a CBM was obtained for 18 healthy individuals, 12 individuals with prediabetes, and 64 individuals with type 2 diabetes mellitus, separated into groups according to fasting blood glucose and oral glucose tolerance tests. The results showed that the CBM reveals diabetes-associated metabolic alterations in the blood, including changes in the levels of carbohydrates, ketone bodies, eicosanoids, phospholipids, and amino acids, which are consistent with the scientific data available to date. The CBM enabled the separation of diabetic patients according to their metabolic metabotypes, providing both a general overview of their metabolic alterations and detailing their individual metabolic characteristics. It was concluded that the CBM is a precise and clinically applicable test for assessing an individual’s metabolic status in diabetes mellitus for diagnostic and treatment purposes.

## 1. Introduction

One anticipated trend in the evolution of clinical laboratory testing is the introduction of high-throughput analytical technologies, which are employed in the omics sciences, particularly metabolomics [1,2]. However, there are various ways to clinically apply metabolomics technologies. The most evident way is to directly apply a single-subject (N-of-1) study workflow employing omics technology to evaluate an individual’s biomaterial.

In this sense, the multi-omics approach, where the biomaterial of a single subject is examined using different omics techniques simultaneously, is the most recognized [3,4,5,6]. For example, under the Integrated Personal Omics Profiling (iPOP) project, which was launched in 2012 [7], an organism’s normal condition is characterized by combining various omics data with a set of personal factors (stress levels, nutrition, activity, and medical history) [8,9,10]. The Arivale program supported the 100 K person wellness project in 2015 after it was proposed in 2014 [11]. In this program, recommendations for enhancing wellbeing and preventing disease were derived from the data gathered over time about each participant, including genome, metabolome, microbiome, and digital self-measurement data.

The Pioneer 100 Wellness Project (P100) began in 2017 and is based on information from individual activity assessments, clinical testing, metabolomes, proteomes, and microbiomes [12]. The multi-omics approach is a productive means of gathering individual molecular data by utilizing different omics technologies [13,14,15], but its use in medicine is difficult and so progresses slowly due to the complexity of omics measurements. These multi-omics projects emphasize that, in order to boost the reproducibility and reliability of multi-omics data, additional method standardization and enhanced quality control are required [16].

Among omics tests being introduced into clinical practice there are single-subject metabolomics studies, which are typically offered in a laboratory-developed test (LDT) format. LDTs are a subset of in vitro diagnostic (IVD) devices [17,18,19,20]. The US Food and Drug Administration (FDA) defines an LDT as “in vitro diagnostic tests that are manufactured and used in a single laboratory”. By controlling the execution of metabolomics tests through the protocols and standardization actions of a single laboratory, the LDT format circumvents the challenges associated with implementing metabolomics in clinical laboratories [21]. Several metabolomic LDTs, such as Meta UDx™, Meta IMD™, and Meta IMD™Plus, were created by Metabolon Inc in 2018 with the goal of identifying metabolites that cannot be measured by other methods, abnormalities in important metabolic pathways, and the identification of certain genetic illnesses. Another company, Nightingale Health, uses a single-finger-prick blood sample to estimate the “age you are likely to live before falling ill from any of the top 10 diseases” using an IVD device that has been CE-marked [22]. The program calculates healthy years using blood nuclear magnetic resonance (NMR) spectroscopy based on data that has already been gathered on hundreds of thousands of individuals. Therefore, the way to help metabolomics become more widely used in medicine is to use metabolomics assays in the LDT format.

Measuring a limited subset of metabolites can also make it easier to perform a personal metabolomics study. Through the measurement of plasma amino acids using liquid chromatography combined with mass spectrometry, the Ajinomoto Group’s AminoIndex^®^ Cancer Screening (AICS^®^) provides minimally invasive, early cancer screening [23]. The AminoIndex^®^ service is an example of how metabolomics has been successfully implemented in clinical settings by simplifying metabolomic analysis.

**Figure 1:**
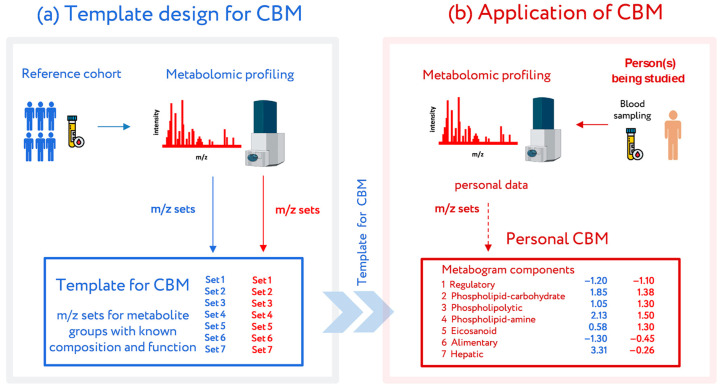
Workflow for the design and application of a clinical blood metabogram (CBM). (**a**) Template design for CBM. A reference cohort of healthy people was used to obtain a template for CBM with defined sets of mass spectrometry picks corresponding to functionally related metabolite groups in the blood. (**b**) Application of the CBM. To use a CBM, sampled blood from a person being studied (or people from a study cohort) is subjected to mass spectrometry after sample preparation to separate the metabolome fraction. The resulting mass peaks are aligned with the characterized sets of mass spectrometry peaks from the template for CBM. To obtain CBM components, their intensities are converted into Z-score scales and averaged over each group. CBM components show the state (normal, upregulated, or downregulated) of the blood metabolome groups, providing in this way clinically relevant information. Adapted from [24].

The most recent attempt to introduce metabolomics into medicine was conducted at the Institute of Biomedical Chemistry (Moscow), where a clinical blood metabogram (CBM) was developed [24]. The CBM is a tailored metabolomics technique that is essentially a condensed N-of-1 metabolomics analysis. Data obtained by direct infusion mass spectrometry (DIMS) of a low molecular fraction of blood, processed by principal component analysis (PCA) and metabolite set enrichment analysis (MSEA), were used to construct the CBM. The CBM avoids the difficulties of every N-of-1 metabolomics investigation and facilitates its future clinical implementation in the LDT format with its quick execution, improved reproducibility, straightforward data processing, and simplified result interpretation (Figure 1).

A wide range of clinically significant data from the blood metabolite groups is displayed in the CBM, including data on humoral regulation, levels of various lipids, amino acids and monosaccharides, data on lipid intake into the body, and liver function. The CBM was previously tested in pilot studies related to obesity and gut microbiota, where its clinical relevance was demonstrated [25,26].

To further describe the clinical capacity of the CBM, it was applied in this work to the most common metabolic disorder, i.e., diabetes mellitus (DM). The diagnostic efficacy, advantages, and limitations of using a CBM in patients with prediabetes and DM were studied and reported.

## 2. Materials and Methods

### 2.1. Subjects

Study participants (*n* = 94) were recruited at the Polyclinic Department of the Endocrinology Research Centre (Moscow, Russia). All participants signed their written informed consent to provide blood samples for research purposes. Blood plasma concentration of diagnostic substances (fasting plasma glucose (FPG), uric acid, total cholesterol, insulin, triglycerides, low-density lipoproteins (LDL), and high-density lipoproteins (HDL)) were measured using an Architect c4000 clinical chemistry analyzer (Abbott Diagnostics, Abbott Park, IL, USA). Glycated hemoglobin (HbA1c) was measured using the Bio-Rad D10 hemoglobin testing system (Bio-Rad Laboratories, France). DM was diagnosed if the FPG level was >5.6 mmol/L.

For the oral glucose tolerance test (OGTT), a standard glucose dose (75 g) was orally ingested, and blood glucose levels were measured two hours later. IGT was diagnosed if the post-load glucose levels were between 7.8 and 11.0 mmol/L (WHO, 1999) [27], and at higher levels, DM was diagnosed.

### 2.2. Mass Spectrometry Analysis of Blood Samples

Venous blood sampling, sample preparation (isolation of blood plasma metabolome by treating with methanol), DIMS analysis using a maXis hybrid quadrupole time-of-flight mass spectrometer (Bruker Daltonics, Billerica, MA, USA) equipped with an electrospray ionization (ESI) source, mass spectra processing, and mass list processing (alignment, standardization, and normalization) were conducted as described previously on the same equipment with the same materials and algorithms [28]. Briefly, blood samples were taken from the vein before the morning meal. The samples (3 mL) were placed into glass tubes containing K_2_EDTA (BD Vacutainer; Becton, Dickinson and Company, Franklin Lakes, NJ, USA) and centrifuged within 15 min of blood collection at 1600× *g* and at room temperature. The resultant blood plasma was subdivided into aliquots that were pipetted into plastic tubes. These tubes were marked, transported in special thermocontainers, frozen, and then stored at −80 °C until analysis. The analyzed samples were subjected to one freeze/thaw cycle.

For plasma deproteinization, aliquots (10 µL) were mixed with 10 µL of water (LiChrosolv; Merck KGaA, Darmstadt, Germany) and 80 µL of methanol (Fluka, Munich, Germany) and incubated at room temperature. After 15 min, the samples were centrifuged at 13,000× *g* (MiniSpin plus centrifuge; Eppendorf AG, Hamburg, Germany) for 10 min. The deproteinized supernatants were then transferred to clean plastic Eppendorf tubes, and fifty volumes of methanol containing 0.1% formic acid (Fluka) were added to each tube. The resulting solutions were subjected to mass spectrometry analysis.

The mass spectrometer was set up to prioritize the detection of ions with a mass-to-charge ratio (*m*/*z*) ranging from 50 to 1000 and a mass accuracy of 1–3 parts per million (ppm). The spectra were recorded in the positive ion charge detection mode. The samples were injected into the ESI source using a glass syringe (Hamilton Bonaduz AG, Bonaduz, Switzerland) connected to a syringe injection pump (KD Scientific, Holliston, MA, USA). The flow rate of the samples to the ionization source was 180 µL/h, and the samples were injected in a randomized order (e.g., control samples were run between case samples). The mass spectra were obtained using DataAnalysis version 3.4 (Bruker Daltonics) to summarize one-minute signals. The ion metabolite masses were determined from the mass spectrum peaks obtained using the DataAnalysis program. All peaks above noise level (signal to noise ratio >1) were selected, and the metabolite ion masses were pooled and processed using the Matlab program (version R2019a; MathWorks, Natick, MA, USA). Alignment of the mass peaks was performed as described previously [29].

### 2.3. Template Design for CBM

**Figure 2:**
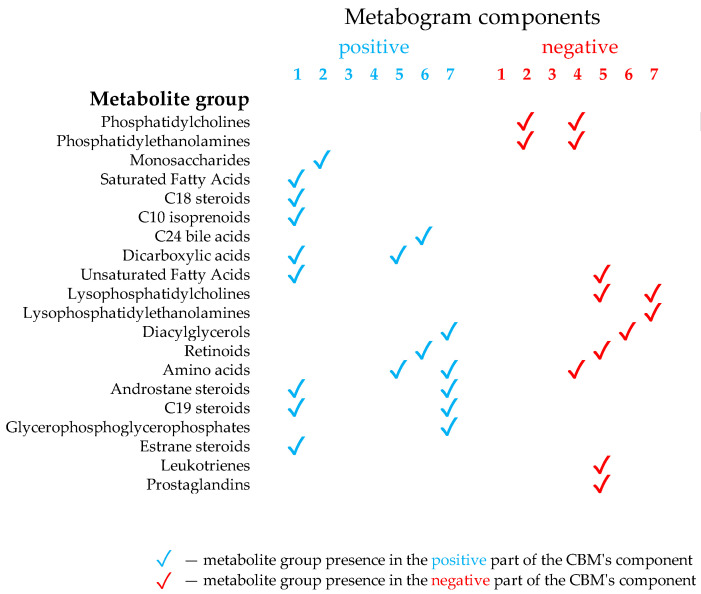
Composition of the clinical blood metabogram components. Adapted from [24].

CBM is a clinical mass spectrometric analysis of a low molecular fraction of blood presented in the form of several values (metabogram components), reflecting the state of the main groups of the blood plasma metabolome with an established composition and clinical significance [24]. The design of the template for the CBM was conducted in previous work using a reference cohort of healthy subjects, and the details of this are described in [24]. Briefly, the blood plasma samples of healthy subjects were analyzed using DIMS (Figure 1). After data preprocessing (alignment, standardization, and normalization), the resulting lists of mass peaks were analyzed by PCA. The sets of mass peaks corresponding to the highest positive or lowest negative principal component coefficients (loadings) were referred to as the blood metabolome components (BMCs). The first seven BMCs, explaining approximately 70% of the blood metabolome variance, formed the CBM’s components. Applying MSEA, the composition of metabogram components was determined by identifying the metabolite classes with which they are enriched (Figure 2). To clarify the biological specificity of the metabogram components, their relationship with the results of the clinical laboratory tests was revealed. Since the principal components have positive and negative coefficients (loadings) involved in the formation of the metabogram components, each metabogram component has two Z-score scales reflecting their measure, called the ‘positive’ and ‘negative’ parts, respectively. The Z-score is a common way of representing data on a unitless scale and is the raw score minus the population mean, divided by the population standard deviation. With a normal distribution, the Z-score is connected to the *p*-values; for example, 1.64 corresponds to *p* = 0.05 (one-tailed), which is thought to be the cutoff for statistical significance and enables the detection of the sample’s deviation from the population. Z-scores of the metabogram components in the −1.64 to +1.64 range are in the normal range; up- and downregulation correspond to higher and lower Z-score values, respectively. Calculations were carried out using the Matlab program.

The components of the CBM reflect the functionally related groups of the blood metabolites associated with humoral regulation (component 1, called ‘regulatory’), the lipid–carbohydrate metabolism (component 2), phospholypolysis (component 3, called ‘phospholipolytic’), the lipid–amine metabolism (component 4), oxidized fatty acids (component 5, called ‘eicosanoid’), the lipid intake into the organism (component 6, called ‘alimentary’), and liver function (component 7 called ‘hepatic’), thereby providing clinically relevant information.

### 2.4. CBM for Participants in the Study Cohort

To obtain the personal CBM of patients, the personal metabolic data are pasted into the template for the CBM. Personal CBMs, which are in fact the prototype of clinical laboratory testing, were obtained using the study cohort (see Section 2.1), which consisted of normal, prediabetic, and diabetic subjects. The mass lists were standardized, normalized, and then aligned with the *m*/*z* values of the template (i.e., with seven *m*/*z* sets corresponding to seven components of the template developed using the reference cohort (see Section 2.3)). Then, the Z-scores for the metabogram components, reflecting the increase or decrease in the concentration of metabolites comprising them, were calculated using the mass peak intensities (by averaging the Z-scores for peaks belonging to the same CBM component) [24].

### 2.5. Adjustment of Mass Peak Intensity Due to Ionic Inconsistency in Samples

The Pearson correlation coefficient was calculated between the aligned and standardized intensities of the mass spectrometry peaks and the FGP level. Intensities that had a correlation coefficient of more than 0.55 were multiplied by 12 (empirically established parameters). This transformation is acceptable since the intensities of peaks belonging to both the experimental and control spectra were multiplied by the coefficient. That is, the data from the experimental samples were not distorted in relation to the control samples.

### 2.6. Cluster Analysis

To provide an overview of CBM types demonstrating deviations in the blood metabolome of the studied subjects, a cluster analysis was carried out. Euclidian distances between metabograms (their Z-scores) were calculated using the *pdist* function (Matlab). A hierarchical cluster tree was generated by the *linkage* function using the ‘ward’ algorithm for computing the distance between clusters. The *dendrogram* function was used to plot the dendrogram.

### 2.7. Diagnostic Parameters

To assess the diagnostic potential of the CBM for prediabetic and diabetic patients, the following diagnostic parameters were evaluated: sensitivity—the percentage of correctly identified positive results (the illness is correctly assigned to prediabetic or diabetic patients); specificity—the percentage of correctly identified negative results (the illness is correctly not assigned to control patients); and accuracy—the percentage of correctly identified positive and negative results.

## 3. Results

### 3.1. Studied Subjects

Ninety-four volunteers—18 healthy, 12 with prediabetes diagnosed by the presence of IGT, and 64 with type 2 DM—were selected for the study. Table 1 presents the clinical characteristics of the cohort. The individual characteristics of the subjects are presented in Supplementary Table S1. The FPG level and OGTT results were used to establish gender-matched case and control groups. The DM group was split into four subgroups:

Group 1: subjects with a negative FPG test and a positive OGTT for diagnosing DM (glucose level > 11.0 mmol/L).

Group 2: subjects with a positive FPG test and a negative OGTT.

Group 3: subjects with a positive FPG test and a positive OGTT for diagnosing IGT (glucose level between 7.8 and 11.0 mmol/L).

Group 4: subjects with a positive FPG test and a positive OGTT for diagnosing DM (glucose level > 11.0 mmol/L).

Therefore, group 1 included subjects with the rare situation when OGTT diagnoses DM while the FPG level is normal. Groups 2 to 4 correspond to DM diagnosed by FPG level at different OGTT results.

### 3.2. CBM Data

**Figure 3:**
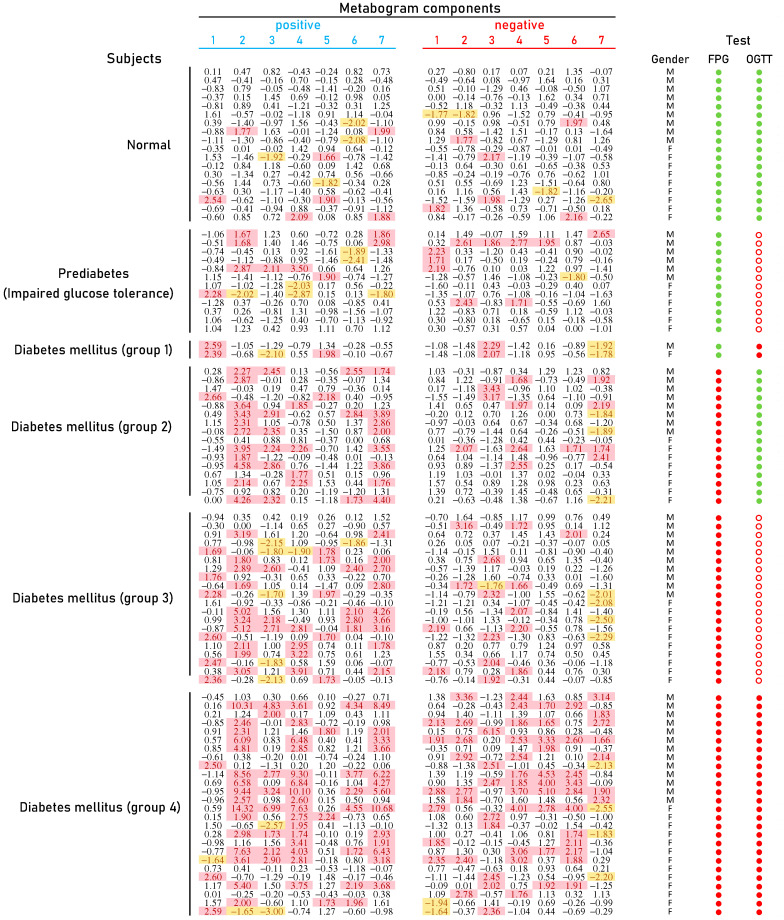
Clinical blood metabogram (CBM) data for control subjects and subjects with prediabetes diagnosed by presence of impaired glucose tolerance (IGT) and with type 2 diabetes mellitus (DM). The data represent the Z-scores of the metabogram components for the individuals. ‘Positive’ values from 1 to 7 and ‘negative’ values from 1 to 7 correspond to two Z-score scales (see Section 2.3). Z-score is a measure of the metabogram components (from −1.64 to +1.64 is the normal range; up- and downregulation correspond to higher and lower Z-score values, respectively). Color coding: red indicates the upregulation of metabolites corresponding to the metabogram component; yellow indicates the downregulation of metabolites corresponding to the metabogram component. ‘M’, male; ‘F’ female; FPG, fasting plasma glucose; OGTT, oral glucose tolerance test; (●) FPG < 5.6 mmol/L and glucose in OGTT < 7.8 mmol/L; (●) FPG > 5.6 mmol/L and glucose in OGTT > 11.0 mmol/L; (○) glucose in OGTT between 7.8 and 11.0 mmol/L.

The typical mass spectra of the low molecular fraction of blood plasma were obtained by direct mass spectrometry. Up to about *m*/*z* 600, peaks of metabolites of various classes were observed, and above *m*/*z* 600, intense peaks of various phospholipids were observed. On average, 8608 peaks were detected in a spectrum. The aligned and standardized mass lists are presented in Supplementary Table S2. These mass spectrometry data were used to obtain personal CBMs for subjects participating in the study (Figure 3).

**Figure 4:**
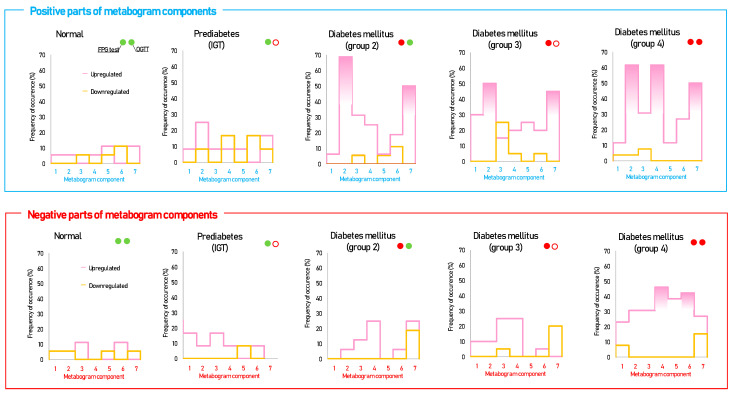
The frequency of deviations from the norm in the clinical blood metabogram (CBM) components for subjects with type 2 diabetes mellitus and prediabetes diagnosed by the presence of impaired glucose tolerance (IGT). The metabogram component deviates from the norm if its Z-score is less than −1.64 (the metabolites composing the metabogram component are downregulated) or above +1.64 (the metabolites composing the metabogram component are upregulated). Frequencies above 40% are filled in red. FPG, fasting plasma glucose; OGTT, oral glucose tolerance test; (●) FPG < 5.6 mmol/L and glucose in OGTT < 7.8 mmol/L; (●) FPG > 5.6 mmol/L and glucose in OGTT > 11.0 mmol/L; (○) glucose in OGTT between 7.8 and 11.0 mmol/L.

Figure 3 shows that patients with DM have deviations in their metabogram components more frequently than control individuals. Several components deviate from the norm more often. For example, the most frequent is metabolite upregulation, reflected by positive components 2 and 7 in DM groups 2–4, as well as by component 4 in DM group 4 (Figure 4).

### 3.3. Statistical Data and Diagnostic Parameters

The *t*-test results indicating the significance of the differences for the metabogram components in the case–control comparison are displayed in Table 2. Diagnostic parameters enabling the evaluation of diagnostic value for the metabogram components are shown in Table 3. This table presents only data on the upregulation of metabogram components, as valuable data on downregulation were not received. In the case–control comparison, the positive components 2 and 7 demonstrate the highest statistical significance as well as the highest diagnostic performance.

### 3.4. Cluster Analysis

**Figure 5:**
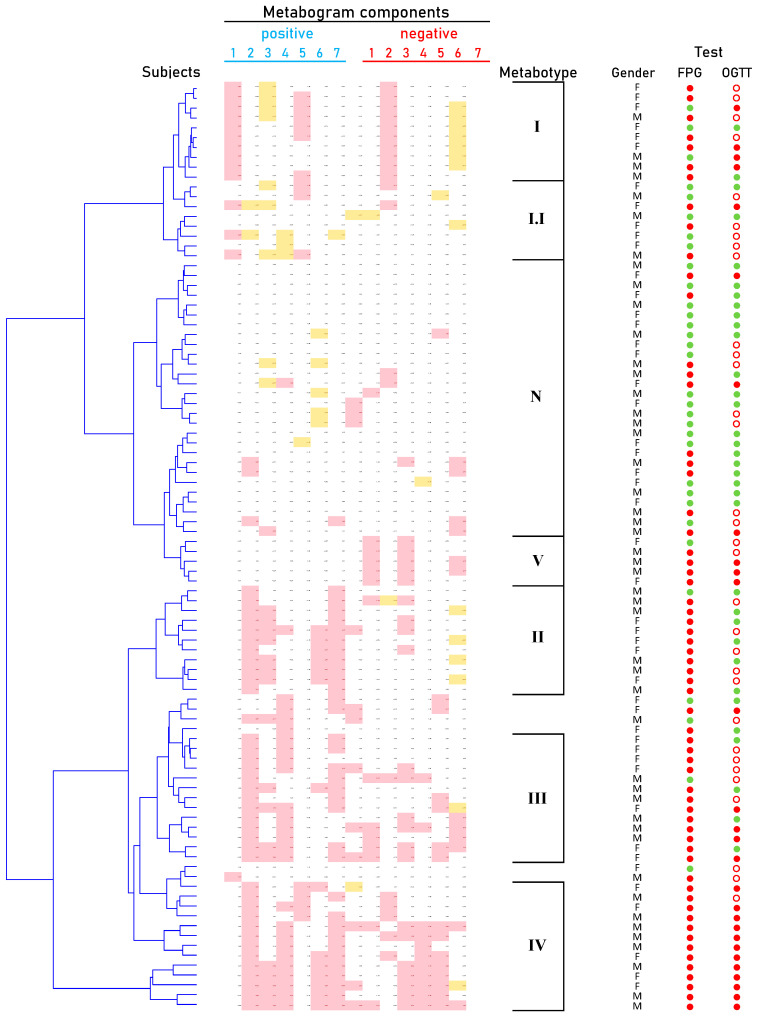
Dendrogram of the clinical blood metabogram (CBM) data of normal subjects and patients with type 2 diabetes mellitus (DM) and prediabetes diagnosed by the presence of impaired glucose tolerance (IGT). Each row corresponds to the Z-scores of the metabogram components for an individual. Z-score is a measure of the metabogram components (from −1.64 to +1.64 is the normal range; up- and downregulation correspond to higher and lower Z-score values, respectively). ‘Positive’ values from 1 to 7 and ‘negative’ values from 1 to 7 correspond to two Z-score scales (see Section 2.3). Color coding: red indicates the upregulation of metabolites corresponding to the metabogram component; yellow indicates the downregulation of metabolites corresponding to the metabogram component. ‘M’, male; ‘F’ female; ‘N’, normal metabotype; FPG, fasting plasma glucose; OGTT, oral glucose tolerance test; (●) FPG < 5.6 mmol/L and < 7.8 mmol/L in OGTT; (●) FPG > 5.6 mmol/L and > 11.0 mmol/L in OGTT; (○) glucose level between 7.8 and 11.0 mmol/L in OGTT.

**Figure 6:**
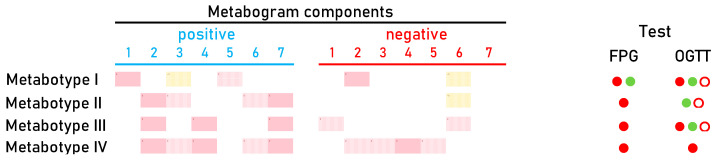
Main types of clinical blood metabograms (CBM) in patients with type 2 diabetes mellitus (DM) and prediabetes diagnosed by the presence of impaired glucose tolerance (IGT). Each row corresponds to the Z-scores of the metabogram components specific for the metabotype. ‘Positive’ values from 1 to 7 and ‘negative’ values from 1 to 7 correspond to two Z-score scales (see Section 2.3). Z-score is a measure of the metabogram components (from −1.64 to +1.64 is the normal range; up- and downregulation correspond to higher and lower Z-score values, respectively). Color coding: red indicates the upregulation of metabolites corresponding to the metabogram component; yellow indicates the downregulation of metabolites corresponding to the metabogram component; the shaded color refers to the metabogram components reflecting optionally up- or downregulated metabolites in DM. FPG, fasting plasma glucose; OGTT, oral glucose tolerance test; (●) FPG < 5.6 mmol/L and < 7.8 mmol/L in OGTT; (●) FPG > 5.6 mmol/L and > 11.0 mmol/L in OGTT; (○) glucose level between 7.8 and 11.0 mmol/L in OGTT.

Cluster analysis was used to identify patterns formed by metabogram components in DM (Figure 5). Some clusters, formed by different combinations of the most often deviating metabogram components, may be considered typical for DM and referred to DM-specific blood metabotypes (Figure 6).

## 4. Discussion

The idea of the CBM is to streamline single-subject metabolomics studies and bring metabolomics into the clinic. Metabolomics studies are complex, time-consuming, and costly due to the thousands of metabolites present in biological samples. The CBM technique eliminates this difficulty [24]. Groups of related metabolites are processed in the CBM, and the enrichment of these groups with metabolite classes is swiftly estimated by the use of MSEA [30]. Thus, faster group analysis replaces the laborious identification of individual metabolites. Moreover, the reproducibility of the results is higher in group analysis. The majority of individual metabolites fail to meet the low coefficient of variation (CV) demonstrated for CBM components [24]. This study is a continuation of the development of CBM, which consists of revealing its properties when applied to patients with DM.

DM is one of the leading causes of illness, disability, and early mortality worldwide [31,32]. Type 1 DM is defined by the death of pancreatic β-cells by an unidentified autoimmune mechanism, typically leading to absolute insulin deficiency [33]. Type 2 diabetes, which accounts for more than 90% of all diabetes cases, is characterized by a progressive lack of adequate insulin secretion from the β-cells as the result of insulin resistance [34]. To diagnose DM and the preceding prediabetes, a FPG level and OGTT are used. The sensitivity of FPG is 49% and its specificity is 98%, according to the systematic review of available data [35]. OGTT, despite being thought to be the ‘gold standard’ for identifying prediabetes associated with IGT at present, has poor reproducibility [36,37,38]. The source of this is analytical and biological variations associated with the test.

The analytical variation is defined as the laboratory test results’ analytical CV. For the intra-laboratory plasma glucose measurement, the analytical CV is 2.5% [39]. The inter-laboratory bias for plasma glucose is 6–7%. Because the FPG test and OGTT use the same laboratory technique, their plasma glucose analytical CVs are identical. The non-analytical change related to time, known as biological variation (intra-individual variation) of laboratory data, is the bigger problem. Glucose demonstrates considerable biological variation [35], with a CV ranging from 4 to 29% [40,41,42,43,44,45,46]. The spread in CV values in healthy individuals has minimal values, while in diabetic patients the CV is significantly higher. Thus, testing modern approaches like CBM, which allows for the panoramic assessment of a person’s metabolites, including carbohydrates, and detailing the individual course of DM with an analytical and biological CV acceptable for clinical laboratory practice (equal to 1.8% and 10.8% [24], respectively, for CBM), is the actual task.

According to the data obtained, in terms of the frequency of occurrence, DM-specific changes in the CBM can be attributed to the upregulation of blood metabolites indicated in the positive parts of components 2, 7, and 4.

*Upregulation of metabolites indicated in the positive part of component 2.* Component 2 is called a ‘phospholipid-carbohydrate’, a positive part of which formed by data from blood carbohydrates (monosaccharides) (Figure 2). Since glucose is the main monosaccharide in the blood and its level is an indicator of IGT and DM, the upregulation indicated in this component was expected and is the main sign of DM in the CBM. The diagnostic accuracy of this component for DM is high—82% for DM group 2 at a sensitivity of 69% and a specificity of 94% (Table 3). Component 2 explains 11.9% of the blood metabolome variance that makes deviation in this component the main one in DM.

*Upregulation of metabolites indicated in the positive part of component 7.* Component 7 is called ‘hepatic’, a positive part of which is formed by data on blood diacylglycerols, glycerophosphates, amino acids, and steroids and is closely related to liver function (Figure 2). The diagnostic accuracy of this component for DM is 71% for DM group 2 at a sensitivity of 62% and a specificity of 94% (Table 3). The connection of this component with DM can be explained by the strong association between insulin resistance and liver pathology, which is highly reproducible in many studies. Hepatic steatosis, or nonalcoholic fatty liver disease (NAFLD), is present in about 70% of type 2 diabetics [47,48] and nearly all obese type 2 diabetics [49] and is a particularly strong predictor of insulin resistance [50,51].

*Upregulation of metabolites indicated in the positive part of component 4.* Component 4 is called ‘phospholipid-amine’ because its negative part reflects the co-directed changes in phospholipids and amino acids in the blood (Figure 2). Unfortunately, metabolites associated with the positive part of this component were not identified during the CBM design [24]. The list of molecular weights for which potential candidates exist was sparse and included several quasi-ions, as presented in Table 4.

The elemental composition of C_2_H_2_O_4_ in the metabolite database corresponds only to oxalic acids. In DM, oxalate is thought to cause kidney damage [52]. Glyoxylate, the precursor of oxalate, has been identified through metabolomic profiling of human plasma to be a potential metabolite marker of DM [53,54]. For the elemental formula C_4_H_6_O_3_, among the candidates are metabolites related to the butanoate metabolism pathway, such as acetoacetic acid (ketone body) and succinic acid semialdehyde. For C_5_H_6_O_5_, there is no alternative to oxoglutaric acid, which also belongs to the butanoate metabolism pathway. Interestingly, all ketone bodies relate to this pathway. DM is the most common pathological cause of elevated blood ketones [55]. In diabetic ketoacidosis, high levels of ketones are produced in response to low insulin levels and high levels of counterregulatory hormones. Thus, it can be assumed that this component of the CBM reflects diabetic ketoacidosis. Since the metabolites of this metabogram component were not reliably identified either according to metabolomics standards or by MSEA during the design of the CBM, the connection of this component with the butanoate metabolism pathway is hypothetical. The diagnostic accuracy of this component for DM group 4 is 75% at a sensitivity of 62% and a specificity of 94% (Table 3).

Apart from the main alterations observed in the CBMs at DM, other more rare changes seen in DM group 4 are observed, such as the upregulation of metabolites indicated in the negative part of components 4, 5, and 6.

*Upregulation of metabolites in DM group 4, indicated in the negative part of component 4.* As it was mentioned before, component 4 is called the ‘phospholipid-amine’ because its negative part reflects the co-directed changes in phospholipids and amino acids (Figure 2). An increase in phospholipids and amino acids in prediabetes and DM due to insulin resistance is an established fact and has been described in many studies, including metabolomic studies [56,57]. The upregulation reflected in this component may be related to this.

*Upregulation of metabolites in DM group 4, indicated in the negative part of component 5.* This metabogram component is called ‘eicosanoid’ due to enrichment with this class of metabolites (Figure 2). Eicosanoids are lipid-derived mediators of inflammation that take place in DM [58,59,60], and their elevation in blood may be reflected in this component.

*Upregulation of metabolites in DM group 4, indicated in the negative part of component 6*. This metabogram component, called ‘alimentary’, is enriched with diacylglycerols (Figure 2). Diacylglycerols are well-studied putative mediators of lipid-induced hepatic insulin resistance [61]. Diacylglycerol species are significantly increased in high-HOMA-IR humans [62] which is consistent with the detected upregulation in this metabogram component.

The distribution of CBM into groups using cluster analysis made it possible to identify the main four metabotypes in DM (Figure 6). Metabotypes II–IV can be assessed as diabetic, as they are associated with an increased level of carbohydrates in the blood, reflected by the positive part of component 2. Of these, metabotype IV can be assessed as a metabotype that, in addition to the main deviations, also has additional ones. In patients with this metabotype, both the FPG test and OGTT are positive (Figure 6). It is known that a positive value for both tests reliably detects DM, which is consistently confirmed by repeated tests over time [63]. Therefore, a similar reliability of the association with DM can be expected for this metabotype.

Metabotype I is different from all the others, since it does not have the typical signs of DM, including the absence of the main characteristic—deviations in carbohydrates. This metabotype is closest to the normal metabotype (metabotype I.I is transitional between them). FPG and OGTT give different results for patients with this metabotype (Figure 6). This metabotype is characterized by deviations in the content of free fatty acids and steroids, the upregulation of which is reflected in the positive part of component 1. There are various ways in which steroids might raise blood sugar levels. They may increase the amount of glucose released by the liver, prevent muscle and fat cells from absorbing glucose from the blood, and lessen the body’s sensitivity to insulin. Any of these factors could indicate that the blood contains too much glucose and may lead to DM [64,65]. DM is also associated with increased total plasma free fatty acid [66]. Therefore, changes in this component of the metabogram are consistent with the behavior of steroids and fatty acids in the development of DM.

The upregulation and downregulation of metabolites characteristic for metabotype 1 and reflected by negative and positive parts of component 3 (the phospholipolytic component), respectively, are quite consistent with the literature data. It is known that the activity of phospholipases is modulated in DM [67,68]. This may reflect increased and decreased levels of phospholiposis substrates and products. The positive part of component 5, which represents the upregulation of certain metabolites such as amino acids and/or dycarboxylic acids (Figure 2), is also in line with findings from science. Increased dicarboxylic acids have been well known in patients with diabetic ketoacidosis [69]. The association of amino acid levels with DM has been described in numerous studies [56,70,71,72,73]. And finally, for metabotype I, metabolite downregulation reflected by a negative component 7 (Figure 6) can be referred to as lysophosphatidylcholines, which are significantly lower in the serum of diabetic patients [74].

After an overview of the metabolic changes associated with metabotype I, it is reasonable to conclude that this metabotype is linked to blood glucose-affecting conditions, which can potentially be attributed to a prediabetic state. This may explain why the results of clinical testing (FPG test and OGTT) in this group of patients differ, indicating both DM and normal status (Figure 6).

Metabotype V is considered a variant of the norm based on its proximity to it and the absence of typical signs of the diabetic metabotype. A distinctive feature is the upregulation of phospholipids in the blood of patients. Moreover, most patients from this small group have positive OGTT and FPG tests. This metabotype shows how metabolomic analysis refutes FPG and OGTT data, thereby providing a reason to adjust existing or make new clinical decisions. The diagnosis of these patients and their treatment must take into account the metabolic profile of their blood.

**Figure 7:**
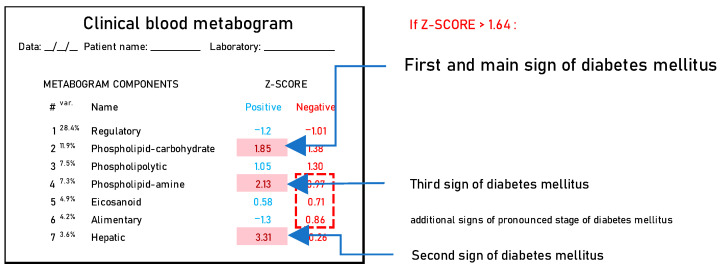
A clinical blood metabogram with diabetes mellitus-associated changes. The percentage of blood metabolome variance that the metabogram component explains is indicated by the superscript “Var.” The metabogram components are measured by the Z-score value, whose normal range is from −1.64 to 1.64. Higher and lower Z-scores are related to the up- and downregulation of the blood metabolites corresponding to the metabogram component. Components of the metabogram that are predominantly altered in diabetes mellitus are highlighted in red. The dashed line highlights non-main changes in diabetes.

Summarizing the DM-associated changes in the CBM, it can be noted that they reflect the main currently known diabetic changes in blood metabolites. Therefore, the CBM can be considered a tool for a panoramic overview of diabetic blood changes, revealing their individual nature and severity. Based on the data obtained, it is possible to propose a DM signature in the CBM (Figure 7).

Based on the results of this study, the benefits that a CBM offers in DM can be highlighted:

- CBM is a new method for diagnosing DM that can complement the FPG level and OGTT;
- CBM makes it possible to identify the metabotypes (molecular phenotypes) of diabetic patients and the deviations in blood metabolites associated with these metabotypes;
- CBM provides precise measurement of diabetic changes in blood metabolome, both in volume and severity;
- Treatment for DM can be tailored to the patient’s needs by means of the CBM’s ability to measure and interpret variations in the blood metabolome. The effectiveness of treatment can be assessed and adjusted by tracking changes in the metabogram components and their return to normal Z-scores. The same applies to assessing lifestyle, selecting an effective diet, physical activity, and other factors affecting health.

Regarding limitations, a CBM cannot reveal IGT diagnosed by OGTT at a normal FPG level (Figure 3 and Table 3). A probable reason for this is that the OGTT is a loading test, and without glucose loading in the blood metabolome there is no evidence of IGT.

The second limitation is that a CBM cannot be applied directly to diabetic patients. Electrolyte disturbances often occur in people with DM. Among them, there is a noticeable deficiency of phosphates, magnesium, and potassium. The incidence of this are even higher in patients with acute diabetic complications [75,76]. ESI, used to obtain data for CBM, is a soft-ionization method that generates ions for use in mass spectrometry. Ions detected by ESI-mass spectrometry are quasimolecular ions that are produced when a cation, such as a proton (a hydrogen ion), potassium, or sodium, is added to the detecting substance. The mass of the additional ion affects the measured mass in each scenario. Consequently, concentrations of these ions in samples should be constant, or at least within a small range, in the case of ESI mass spectrometry. To obtain reliable results for patients with DM, changes associated with ionic inconsistency should be leveled out in mass spectrometry data, e.g., as was performed in this work (see Section 2.5).

It should also be noted that the template for CBM was formed from experimental data and therefore depends on the protocols used. Despite the fact that the metabogram components reflect the main processes in the body, which makes them versatile, the dependence on the measurement protocols makes the template quite biased. Templates obtained from different laboratories may have differences until one template is accepted as a reference and deposited in the widely used public database. Also, to date, the influence of genome, gender, age, circadian, and other rhythms on CBM has not been studied, which may be a source of errors in the interpretation of CBM.

The further development of CBM and its combination with dried blood spot (DBS) samples [77] may offer additional opportunities for using CBM in laboratory diagnostics by enabling the unassisted collection of capillary blood at home. Given the prevalence of DM, it is especially necessary to make CBM testing accessible and convenient for clients through mail-order transportation of DBS samples to the laboratory. It will also be of interest to explore the potential of CBM in a multi-omics approach, for example, by combining a CBM with blood plasma proteomic data. It is quite possible to carry out proteomic analysis in accordance with the concept of a metabogram, that is, to conduct a quick group analysis of blood proteins. Blood analysis at different omic levels will allow for a more complete picture of blood changes in DM.

## 5. Conclusions

It was shown that CBM has clinical value for diabetic individuals by revealing DM-related metabolic alterations in the blood. Being an omics test, CBM provides a broad overview of these alterations, thereby providing an individual metabolic picture of the course of DM in a particular patient. A CBM reflects not only the degree of these alterations but also their significance for the body’s functioning. A CBM allows for the classification of metabolic alterations to reveal DM-specific metabotypes that provide a basis for tailoring diabetic patient treatment. Therefore, further testing of CBM as an LDT and its incorporation into clinical practice is justified.

## Figures and Tables

**Table 1:** Study cohort characteristics.

Parameter	Groups					
	Normal	Prediabetes	Type 2 Diabetes Mellitus			
			Group 1	Group 2	Group 3	Group 4
Number	18	12	2	16	20	26
Gender (males/females)	9/9	6/6	1/1	8/8	10/10	13/13
Age (years)	54.7 ± 13.2 ^1^	63.1 ± 9.6	63, 81	48.8 ± 12.5	54.6 ± 12.0	58.8 ± 11.0
BMI (kg/m^2^)	33.4 ± 9.1	35.7 ± 10.1	28.7, 38.9	33.7 ±8.9	33.5 ± 6.2	33.6 ± 6.8
FPG (mmol/L)	5.3 ± 0.3	5.4 ± 0.2	5.1, 5.6	6.2 ± 0.4	7.1 ± 1.7	8.0 ± 2.3
Glucose in OGTT (mmol/L)	6.2 ± 0.9	9.3 ± 0.9	14.1, 11.4	6.2 ± 1.3	9.2 ± 0.9	14.3 ± 2.3
HbA1c (%) [mmol/mol]	5.7 ± 0.4	6.1 ± 0.5	6.2, 6.0	5.9 ± 0.5	6.3 ± 0.6	7.2 ± 1.5
LDL (mmol/L)	3.5 ± 0.8	3.2 ± 1.0	2.7, 2.8	3.3 ±0.9	3.5 ± 1.1	3.6 ± 1.0
HDL (mmol/L)	1.2 ± 0.4	1.2 ± 0.4	0.7, 1.4	1.1 ±0.2	1.1 ± 0.3	1.0 ± 0.3
Triglycerides (mmol/L)	1.3 ± 0.5	1.3 ± 0.4	2.1, 0.8	1.4 ±0.6	2.5 ± 2.9	2.7 ±2.1
Cholesterol (mmol/L)	5.2 ± 0.9	4.9 ± 1.2	4.7, 4.5	5.1 ± 0.9	5.6 ± 1.1	5.7 ± 1.4
Uric acid (µmol/L)	386 ± 82	392 ± 97	380, 266	353 ± 94	395 ± 97	356 ± 88
Insulin (µU/mL)	15.2 ± 22.6	13.4 ± 7.7	12.1, 5.8	15.9 ± 13.4	26.9 ± 31.8	25.1 ± 33.3
HOMA-IR	3.6 ± 5.4	3.2 ± 1.8	2.7, 1.4	4.3 ± 3.4	8.6 ± 9.8	8.3 ± 9.5

^1^ mean ± standard deviation. FPG, fasting plasma glucose; OGTT, oral glucose tolerance test; IGT, impaired glucose tolerance; HOMA, homeostatic model assessment = fasting glucose x insulin / 22.5; HbA1c, hemoglobin A1c test; LDL, low-density lipoproteins; HDL, high-density lipoproteins; BMI, body mass index.

**Table 2:** Statistical significance for the separation of subjects with prediabetes and diabetes mellitus (DM) from normal subjects based on clinical blood metabogram (CBM) data.

Metabogram Component		*t*-Test (*p*-Value)				
		Normal Subjects Versus Subjects with				
		Prediabetes ^1^	Type 2 Diabetes Mellitus			
			Group 2	Group 3	Group 4	All Groups
Positive	1	0.67	0.83	0.01	0.33	0.13
	2	0.79	0.00001	0.008	0.0006	0.0008
	3	0.72	0.01	0.71	0.09	0.11
	4	0.39	0.11	0.04	0.0003	0.01
	5	0.64	0.73	0.04	0.09	0.15
	6	0.19	0.05	0.25	0.04	0.04
	7	0.93	0.0003	0.003	0.0006	0.0005
Negative	1	0.36	0.10	0.80	0.05	0.17
	2	0.65	0.52	0.70	0.02	0.13
	3	0.46	0.97	0.08	0.24	0.26
	4	0.34	0.006	0.17	0.001	0.004
	5	0.64	0.54	0.28	0.0001	0.01
	6	0.53	0.37	0.19	0.002	0.02
	7	0.83	0.92	0.03	0.95	0.53

^1^ Prediabetes was diagnosed by the presence of impaired glucose tolerance. ‘Positive’ and ‘negative’ correspond to two parts of the Z-score scales, which are the measure of the CBM components (see Section 2.3).

**Table 3:** Parameters for diagnosing prediabetes and type 2 diabetes mellitus by identifying upregulated blood metabolites in a clinical blood metabogram (CBM).

Metabogram Component		${Accuracy\left(\%\right)}_{Specificity(\%)}^{Sensitivity(\%)}$			
		Prediabetes ^1^	Type 2 Diabetes Mellitus		
			Group 2	Group 3	Group 4
Positive	1	$60_{94}^{8}$	$53_{94}^{6}$	$61_{94}^{30}$	$45_{94}^{12}$
	2	$67_{94}^{25}$	$82_{94}^{69}$	$71_{94}^{50}$	$75_{94}^{62}$
	3	$63_{100}^{8}$	$71_{100}^{38}$	$55_{100}^{15}$	$59_{100}^{31}$
	4	$60_{94}^{8}$	$62_{94}^{25}$	$55_{94}^{20}$	$75_{94}^{62}$
	5	$57_{89}^{8}$	$50_{89}^{6}$	$55_{89}^{25}$	$43_{89}^{12}$
	6	$60_{100}^{0}$	$62_{100}^{19}$	$58_{100}^{20}$	$57_{100}^{27}$
	7	$60_{89}^{17}$	$71_{89}^{50}$	$66_{89}^{45}$	$66_{89}^{50}$
Negative	1	$67_{94}^{25}$	$50_{94}^{0}$	$50_{94}^{10}$	$52_{94}^{23}$
	2	$63_{94}^{17}$	$53_{94}^{6}$	$50_{94}^{10}$	$57_{94}^{31}$
	3	$60_{83}^{25}$	$50_{83}^{13}$	$53_{83}^{25}$	$52_{83}^{31}$
	4	$67_{100}^{17}$	$65_{100}^{25}$	$61_{100}^{25}$	$68_{100}^{46}$
	5	$63_{100}^{8}$	$53_{100}^{0}$	$47_{100}^{0}$	$64_{100}^{48}$
	6	$53_{89}^{0}$	$50_{89}^{6}$	$45_{89}^{5}$	$61_{89}^{42}$
	7	$63_{100}^{8}$	$65_{100}^{25}$	$47_{100}^{0}$	$57_{100}^{27}$

^1^ Prediabetes was diagnosed by the presence of impaired glucose tolerance. ‘Positive’ and ‘negative’ correspond to two parts of the Z-score scales reflecting the measure of CBM components (see Section 2.3). Accuracy greater than 70 percent and the corresponding sensitivity and specificity are highlighted in red.

**Table 4:** Metabolite ions from the positive part of component 4 of the template for CBM for which the candidate metabolites exist in the metabolite database.

Measured *m*/*z*	Calculated *m*/*z*	Delta Δ*m*/*z*	Detected Ion	Elemental Formula
128.963	128.9585	0.0045	[M+K^39^]^+^	C_2_H_2_O_4_
140.995	140.9949	0.0001	[M+K^39^]^+^	C_4_H_6_O_3_
142.990	142.9930	−0.0030	[M+K^40^]^+^	C_4_H_6_O_3_
184.985	184.9847	0.0003	[M+K^39^]^+^	C_5_H_6_O_5_

## Data Availability

The data presented in this study are available as Supplementary Materials and on request from the corresponding author.

## Supplementary Materials

The following are available online at https://www.mdpi.com/article/10.3390/metabo14030168/s1, Table S1: Clinical test results for studied subjects. Table S2: Aligned and standardized mass lists.
